# Robotic pyelolithotomy for staghorn nephrolithiasis during partial nephrectomy

**DOI:** 10.1590/S1677-5538.IBJU.2015.0282

**Published:** 2016

**Authors:** Hiury S. Andrade, Homayoun Zargar, Peter A. Caputo, Oktay Akca, Daniel Ramirez, Onder Kara, Robert J. Stein, Jihad H. Kaouk

**Affiliations:** 1 Center for Robotic and Image Guided Surgery, Glickman Urological and Kidney Institute, Cleveland Clinic, Cleveland, OH, USA

## INTRODUCTION

Although the incidences of kidney cancer and urolithiasis are increasing (1, 2) the discovery of both pathologies in the same patient is uncommon. This video demonstrates the simultaneous management of a staghorn calculus and an ipsilateral renal mass using the robotic platform.

## CASE

A 68-year-old woman was diagnosed with a 3.9cm left partial staghorn calculus and a 3.0x2.7cm left upper pole renal mass after an acute left flank pain episode. The patient had a history of hypertension, hyperlipidemia, coronary artery disease, asthma, hypothyroidism and obesity (BMI 39Kg/m^2^).

Intraoperatively after colon mobilization and hilum dissection, the Gerota’s fascia was incised and the entire surface of the kidney was exposed. The ureter was carefully dissected up to the renal pelvis. Intraoperative ultrasound identified the stone location and delineated the tumor borders. A posterior pyelotomy was performed using cold scissors and the stone removed in its entirety. A double J stent was inserted in an anterograde manner followed by the pyelotomy closure. The partial nephrectomy was then performed using our standard technique (3).

## RESULTS

The operative time was 240 minutes and the estimated blood loss was 150ml. There were no intra or postoperative complications. Final histopathology showed a T1a renal cell carcinoma, clear cell papillary type with a negative surgical margin. The double J stent was removed after 4 weeks and the patient remains asymptomatic at 3 months postoperatively.

## CONCLUSIONS

Simultaneous robotic pyelolithotomy and partial nephrectomy is a safe and feasible treatment for this uncommon presentation.

## ARTICLE INFO

Available at: **www.intbrazjurol.com.br/video-section/andrade_623_625/**

Int Braz J Urol. 2016; 42 (Video #4): 623-5
