# Supplementary material for: Robotic pyelolithotomy for staghorn nephrolithiasis during partial nephrectomy
Source: Int Braz J Urol. 2016 May-Jun;42(3):623–5. doi: 10.1590/S1677-5538.IBJU.2015.0282 (PMC4920585; doi:10.1590/S1677-5538.IBJU.2015.0282)
Supplement: Related texts

# Letter: EDITORIAL COMMENT

**Authors:** Fabio C. M. Torricelli

Simultaneous robot-assisted laparoscopic pyelolithotomy and partial nephrectomy is a reasonable approach for patients suffering from renal stones that are diagnosed with renal cancer (1). In this video, the authors presented a successful procedure for stone and tumor removal through the same surgical access (2-3). This minimally invasive surgery combines the advantages of a laparoscopic pyelolithotomy over a percutaneous nephrolithotomy (lower incidence of bleeding and higher stone-free rate) with the benefits of a robotic procedure for the treatment of a renal cancer (lower warm ischemia time). This surgical approach should be considered as an alternative when treating patients with this uncommon presentation.

Fabio C. M. Torricelli, MDAv. Vereador Jose Diniz, 330 / 208Sao Paulo, SP, 04604-006, BrasilPhone: +55 11 5533-4900E-mail: fabio.torricelli@hotmail.com

## References

[1] Wang X, Li S, Liu T, Guo Y, Yang Z (2013). Laparoscopic pyelolithotomy compared to percutaneous nephrolithotomy as surgical management for large renal pelvic calculi: a meta-analysis. J Urol.

[2] Badalato GM, Hemal AK, Menon M, Badani KK (2009). Current role of robot-assisted pyelolithotomy for the management of large renal calculi: a contemporary analysis. J Endourol.

[3] Aboumarzouk OM, Stein RJ, Eyraud R, Haber GP, Chlosta PL, Somani BK (2012). Robotic versus laparoscopic partial nephrectomy: a systematic review and meta-analysis. Eur Urol.

# Letter: EDITORIAL COMMENT

**Authors:** David J. Hernandez

Management of a kidney containing both an enhancing renal mass and a partial staghorn calculus presents several dilemmas. As an alternative to radical nephrectomy, most urologists would likely perform staged procedures typically involving percutaneous nephrolithotomy and partial nephrectomy. While robotic assisted partial nephrectomy is well accepted for management of the renal mass, robotic lithotomy is infrequently performed for the removal of large stones (1-3). This video by Andrade et al from the Cleveland Clinic nicely demonstrates a robotic pyelolithotomy for a large stone at the time of robotic partial nephrectomy. Though this may be an uncommon situation, the video clearly shows the feasibility and safety of this combined procedure, at least in expert hands. This case avoided multiple surgeries and allowed for complete stone removal and tumor resection while maintaining renal function.

David J. Hernandez, MDDirector, USF Urology Clinic SouthUrologic Malignancies, Robotic Surgery, BPH e UrolithiasisChief of Urology, Tampa General Hospital2 Tampa General Circle, STC 6th floorTampa, FL 33606, USAFax: +1 813 259-8645E-mail: dhernan3@health.usf.edu

## References

[1] Ghani KR, Trinh QD, Jeong W, Friedman A, Lakshmanan Y, Omenon M, Elder JS (2014). Robotic nephrolithotomy and pyelolithotomy with utilization of the robotic ultrasound probe. Int Braz J Urol.

[2] Badalato GM, Hemal AK, Menon M, Badani KK (2009). Current role of robot-assisted pyelolithotomy for the management of large renal calculi: a contemporary analysis. J Endourol.

[3] Kramer BA, Hammond L, Schwartz BF (2007). Laparoscopic pyelolithotomy: indications and technique. J Endourol.
